# Efficacy of butylphthalide in the treatment of patients with stroke attributed to intracranial arterial stenosis

**DOI:** 10.12669/pjms.40.9.10100

**Published:** 2024-10

**Authors:** Xudong Lu, Shuxia Qian, Yanping Wang, Xiaoling Zhang, Yuhua Jin, Bo Yu

**Affiliations:** 1 Xudong Lu, Department of Neurology, Jiaxing Second Hospital, Jiaxing, Zhejiang Province 314000, P.R. China; 2 Shuxia Qian, Department of Neurology, Jiaxing Second Hospital, Jiaxing, Zhejiang Province 314000, P.R. China; 3 Yanping Wang, Department of Neurology, Jiaxing Second Hospital, Jiaxing, Zhejiang Province 314000, P.R. China; 4 Xiaoling Zhang, Department of Neurology, Jiaxing Second Hospital, Jiaxing, Zhejiang Province 314000, P.R. China; 5 Yuhua Jin, Department of Neurology, Jiaxing Second Hospital, Jiaxing, Zhejiang Province 314000, P.R. China; 6 Bo Yu, Department of Neurology, Jiaxing Second Hospital, Jiaxing, Zhejiang Province 314000, P.R. China

**Keywords:** Butylphthalide, Intracranial artery stenosis, Stroke

## Abstract

**Objective:**

To explore the clinical efficacy of butylphthalide in treating patients with stroke attributed to intracranial artery stenosis (ICAS).

**Methods:**

In this retrospective study, records of 163 patients with stroke attributed to ICAS admitted to Jiaxing Second Hospital from January 2021 to January 2023 were retrospectively analyzed. Patients were divided into two groups based on the treatment received: control group (patients received routine treatment, n=55) and observation group (patients treated with butylphthalide on a routine basis, n=58). Changes in levels of cerebrovascular reactivity (CVR), breath-holding index (BHI), pulsatility index (PI), and middle cerebral artery mean flow velocity (Vm) between the two groups before and after treatment were compared. In addition, cognitive function, neurological function, and living ability were compared between the two groups before and after treatment, as well as the overall clinical efficacy of the treatment.

**Results:**

The baseline data was comparable between the two groups (*P*>0.05). After the treatment, CVR, BHI, and Vm indicators in the observation group were significantly higher than those in the control group, while the levels of PI indexes were significantly lower than those in the control group (*P*<0.05). Montreal Cognitive Assessment (MoCA) and Barthel scale scores of the observation group were significantly higher compared to the control group, while the scores of National Institutes of Health Stroke Scale (NIHSS) were significantly lower (*P*<0.05).

**Conclusions:**

Butylphthalide in addition to routine treatment can effectively improve cerebrovascular reserve function, promote neurological and cognitive dysfunction recovery, and enhance daily living ability of patients with stroke caused by ICAS.

## INTRODUCTION

Intracranial artery stenosis (ICAS) is one of the most common causes of ischemic stroke, with an estimated prevalence of 6% in the middle-aged and elderly population.^1^ ICAS is caused by the formation of atherosclerotic plaques, arteritis, muscle fiber dysplasia and other causes of cerebral artery wall lesions and lumen thinning.^2^,^3^ When the cerebral artery lumen narrows to a certain extent, the decreased blood flow to the distal end of the brain through the narrowed artery may lead to tissue ischemia and hypoxia, brain cell death, and ultimately ischemic stroke.^3^,^4^ Ischemic stroke has a sudden onset and often presents with symptoms of brain dysfunction, such as limb numbness, hemiplegia, language disorders, cognitive impairment, etc. It can also lead to varying degrees of neurological deficits, seriously threatening the patient’s life safety and affecting their quality of life.^5^,^6^

While routine drug treatment of ischemic stroke with thrombolytics, blood lipid lowering agents, and anticoagulants. may effectively alleviate symptoms and control the condition, it is easy to overlook potential neurological deficits, which impacts the prognosis if stroke patients.^6^,^7^ Butylphthalein is a new level Class-I drug with anti-inflammatory properties that was shown to protect mitochondria, inhibit thrombosis, and promote cerebral microcirculation remodeling.^8^ Studies have shown that butylphthalide can reconstruct microcirculation in the ischemic zone and plays an important neuroprotective role by improving energy metabolism and other pathways.^8^,^9^ However, there is limited literature on the treatment of stroke caused by intracranial arterial stenosis with butylphthalide. The main purpose of this study was to retrospectively analyze clinical effectiveness of butylphthalide in treating patients with stroke attributed to ICAS.

## METHODS

In this study, we retrospectively reviewed the clinical data of 113 patients (58 males and 55 females) with stroke caused by ICAS who received treatment at Jiaxing Second Hospital from March 2021 to March 2023. Patients were divided into two groups based on the treatment received: control group (patients received routine treatment, n=55) and observation group (patients treated with butylphthalide on a routine basis, n=58).

### Ethical Approval:

The ethics committee of Jiaxing Second Hospital approved this retrospective study with the number 2024-003-01, date: Feb. 04^th^ 2024.

### Inclusion criteria:

- Patients met the diagnostic criteria of ischemic stroke, as confirmed by cranial computer tomography (CT) or magnetic resonance imaging (MRI).^10^
- Concomitant ICAS, with a stenosis rate of ≥ 50% and ≤ 70%.
- Patients with the first onset within 48 hours.
- Patients with no strict center of gravity, liver or kidney organ dysfunction.
- Patients who were conscious enough to cooperate with the examiner.

### Exclusion criteria:

- Patients with concurrent intracranial hemorrhage and malignant tumors.
- Patients with severe infection.
- Patients with a history of drug allergies.
- Pregnant or lactating women.
- Patients with concomitant mental or neurological disorders.
- Patients with concomitant stenosis of other major arteries in the ipsilateral internal carotid artery system.
- Patients who have undergone defibrillation, anticoagulation, and thrombolysis before the treatment.

### Routine treatment:

Individualized treatment was used, and the patient was given oxygen inhalation. Mannitol (manufacturer: Shanghai Baite medical supplies Co., Ltd; China) was used to reduce intracranial pressure and neuroprotective agents were administered to protect brain tissue. Intravenous thrombolysis was done with alteplase (manufacturer: Boehringer-Ingelheim; Germany) (administered at a dose of 0.9mg/kg and a total amount of < 90mg). Briefly, 10% alteplase was mixed with 10ml of physiological saline for intravenous drip, and the remaining 90% alteplase was mixed with 100ml of physiological saline. Continuous intravenous infusion was initiated within 60 minutes. The use of clopidogrel (manufacturer: Sanofi (Hangzhou) Pharmaceutical Co., Ltd; China) and aspirin (manufacturer: Bayer healthcare; Germany) was prohibited 24 hours after the medication. Aspirin was prescribed to inhibit platelet aggregation at a dose of 75-100 mg/d. Atorvastatin (manufacturer: Pfizer Pharmaceuticals Limited; USA) was administered for lipid-lowering (dose 10-20mg/dose/day). Special attention was paid to the prevention and treatment of complications. Treatment was continued for one month.

### Butylphthalein:

On the basis of routine treatment, patients in the observation group were also given 25mg of Butylphthalein Sodium Chloride Injection (manufacturer: Enbipu Pharmaceutical Co., Ltd. of Shiyao Group, approval number: H20100041, specification: 25mg/100ml) and 100ml of physiological saline for intravenous drip treatment. The treatment was carried out twice a day with an interval of more than six hours, and the infusion time was set to ≥ 50 minutes. Treatment was continued for one month.

### Outcome measures:

1) Changes in levels of cerebrovascular reactivity (CVR), breath-holding index (BHI), pulsatility index (PI), and middle cerebral artery mean flow velocity (Vm) were measured using the EXP-9D detector produced by Nanjing Ostai Biotechnology Co., Ltd. for transcranial Doppler (TCD); 2MHz and 4MHz probes were used to simultaneously examine middle cerebral artery on both temporal windows, and the head was fixed with a Spencer stent. After patient’s breathing calmed and the baseline blood flow velocity of the middle cerebral artery on the affected side became stable, the average flow velocity (Vm1) and PI of the middle cerebral artery in the resting state were recorded. The patient held their breath for one minute to induce hypercapnia, and the flow velocity of the middle cerebral artery (Vm2) recorded in the gas state. After resting for 15 minutes, the above experiment was repeated twice and the average value was taken. If there was stenosis in both middle cerebral arteries, the lateral data with significant changes in average flow velocity was taken for statistical analysis. Calculation methods were as follows: BHI=(Vm2-Vm1) × 100%/(Vm1 × Breath holding time);^11^ Vm rise rate (%)=(Vm2-Vm1) × 100%/Vm1;^12^ CVR=[(Vm1-Vm2)/Vm2] × 100%.^13^ CVR less than 10% indicates that the CVR is damaged.

***2) Cognitive function***: Cognitive function of the patients were evaluated using Montreal Cognitive Assessment (MoCA) scale.^14^ This scale includes 11 items in multiple cognitive domains, such as attention and concentration, language, executive and visuospatial function, abstraction, naming, orientation, and memory, with a maximum score of 30 points. A score less than 26 indicates cognitive impairment.

***3) Neurological function:*** The National Institutes of Health Stroke Scale (NIHSS) was used for evaluation of neurological function, with a maximum score of 42.^15^ The higher the score, the more severe the neurological damage.

***4) Living ability:*** Daily living ability of the patients was evaluated using the Barthel index.^16^ The scale has 10 items and is graded based on the patient’s self-care needs, with scores of 0, 5, 10, and 15 out of 100. Higher the score indicates better ability to take care of themselves.

***5) Clinical efficacy was categorized into three levels: a. Significantly effective:*** symptoms and signs disappear, biochemical and laboratory test indicators return to normal; ***b. Effective:*** a significant improvement in symptoms and signs, and biochemical and laboratory test indicators tend towards normal values; ***c. Invalid:*** no improvement or condition worsening. Total effective rate (%) = (cases of significantly effective+effective)/total number of cases × 100%.

### Statistical analysis:

SPSS 22.0 software (IBM Corp, Armonk, NY, USA) was used for analysis. Quantitative data were represented by mean ± standard deviation, independent sample *t*-test was used for inter group comparison, and paired *t*-test was used for intra group before and after comparison. Count data was analyzed using chi square test to represent the number of cases. PRISM8.0 software (GraphPad, San Diego, USA) was used to draw bar charts of MoCA, NIHSS, and Barthel scores. *P*<0.05 was statistically significant. All reported *p*-values were bilateral.

## RESULTS

A total of 113 patients were included in this study, 55 in the control group and 58 in the observation group. Age range of the patients was 60 to 79 years, with an average age of 67.99 ± 4.59 years. Neither group of patients reported any adverse reactions, and the baseline data was comparable between the two groups (*P*>0.05) (Table-I). Before the treatment, there was no significant difference in the levels of CVR, BHI, PI, and Vm between the two groups (*P*>0.05). After the treatment, levels of CVR, BHI, and Vm in the observation group were significantly higher than those in the control group, while the levels of PI were significantly lower than those in the control group (*P*<0.05) (Table-II).

**Table-I:** Comparison of general data between the two groups.

Section	Control Group (n=55)	Observation Group (n=58)	χ^2^/t	p
Male (yes)	25 (45.45)	32 (55.17)	1.066	0.302
Age (years)	67.67±4.42	68.29±4.75	-0.717	0.475
Onset time (hours)	17.55±6.15	18.14±7.54	-0.459	0.647
Total cholesterol (mmol/l)	5.70±0.67	5.58±0.92	0.821	0.413
Hypertension (yes)	29 (52.73)	26 (44.83)	0.705	0.401
Diabetes mellitus (yes)	21 (38.18)	31 (53.45)	2.649	0.104
Hyperlipidemia (yes)	20 (36.36)	30 (51.72)	2.700	0.100
Smoking (yes)	24 (43.64)	31 (53.45)	1.088	0.297
Drink (yes)	19 (34.55)	17 (29.31)	0.356	0.551

**Table-II:** Comparison of CVR, BHI, PI and Vm between two groups (*χ̅*±*S*).

Section	n	CVR (%)		BHI		PI		Vm (cm/s)	

		Before treatment	After treatment	Before treatment	After treatment	Before treatment	After treatment	Before treatment	After treatment
Control Group	55	7.15±1.41	9.95±1.73*	0.70±0.15	0.81±0.19*	0.90±0.10	0.78±0.12*	27.36±2.26	30.98±2.45*
Observation Group	58	7.38±1.36	12.19±2.41*	0.69±0.16	1.13±0.23*	0.92±0.13	0.66±0.15*	26.98±2.13	35.71±3.08*
t	-	0.227	17.247	0.880	14.975	0.557	9.805	0.128	9.414
P	-	0.820	0.000	0.380	0.000	0.578	0.000	0.897	0.000

***Note:***

* Indicates the comparison within the group and before treatment, P < 0.05.

**Fig.1:**
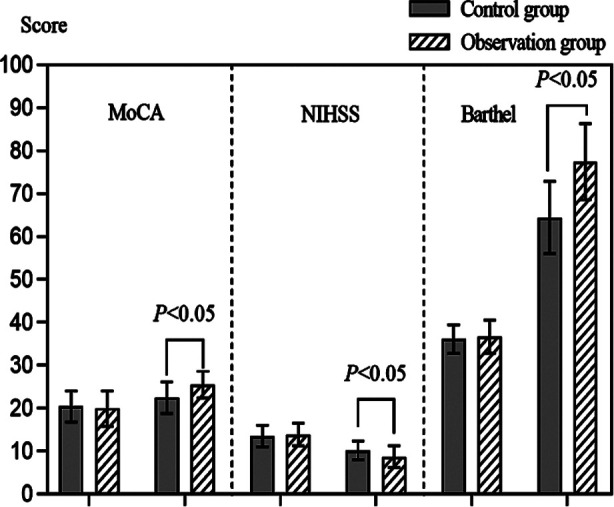
Comparison of MOCA, NIHSS, and Barthel scores before and after the treatment in two groups; Montreal Cognitive Assessment (MoCA); National Institutes of Health Stroke Scale (NIHSS).

Before the treatment, there was no significant difference in MoCA, NIHSS, and Barthel scores between the two groups of patients (*P*>0.05); After the treatment, the MoCA and Barthel scores in the observation group were significantly higher than those in the control group, while the NIHSS scores were significantly lower than those in the control group (*P*<0.05) (Fig.1). Clinical efficacy of the observation group after the treatment was significantly higher than that of the control group (*P*<0.05) (Table-III).

**Table-III:** Comparison of clinical effects between two groups.

Group	n	Effective	Valid	Invalid	Total clinical efficacy
Control group	55	27 (49.09)	20 (36.36)	8 (14.54)	47 (85.45)
Observation group	58	33 (56.89)	24 (41.37)	1 (1.72)	57 (98.27)
*χ* ^2^	-				4.702
*P*	-				0.030

## DISCUSSION

This study showed that in patients with stroke caused by ICAS, treatment with butylphthalide in addition to conventional therapy can effectively improve cerebrovascular reserve function, arterial hemodynamic related indicators, cognitive and neurological function, enhance daily living ability and improve clinical efficacy. Our results are in agreement with previous reports.^17^,^18^

Chen et al.^17^ reported that butylphthalide has therapeutic effect on diabetes, diabetes-induced cataract, atherosclerosis and other non-nervous system diseases. Numerous studies have demonstrated that butylphthalide exerts its pharmacological effect by improving microcirculation and protecting mitochondria, inhibiting oxidative stress, neuronal apoptosis, and inflammatory response, in addition to antiplatelet and anti-thrombotic effects.^8^,^9^,^17^ A systematic review and meta-analysis by Fan et al.^18^ demonstrated in that butylphthalide can improve recovery of cognitive function and daily living ability, and alleviate neurological deficits with no serious adverse reactions. We may speculate that butylphthalide effectively blocks multiple pathological links of brain injury caused by cerebral ischemia through multi-target mechanisms, has a strong anti-ischemic effect, inhibits neuronal apoptosis, protects mitochondrial function, promotes the recovery of neurological deficits, promotes collateral circulation, improves hemodynamics, and reconstructs microcirculation in ischemic brain tissue.^17^,^18^

Studies have shown that CVR is the main criterion for predicting patient prognosis and an independent risk factor for stroke.^19^,^20^ Transcranial Doppler utrasonography can accurately detect changes in intracranial arterial blood flow velocity in real-time, while breath holding test can objectively reflect the intracranial medial branch circulation and automatic regulation function, and to a certain extent reflect the CVR function.^19^,^21^ Our results showed that levels of CVR, BHI, PI, and Vm indexes in the observation group were better than those in the control group, indicating that butylphthalide can effectively improve cerebrovascular reserve of patients.

Moreover, a long-term compensatory dilation of blood vessels can easily lead to a decrease in the level of CVR indicators.^20^ While conventional treatment can improve clinical symptoms of patients to a certain extent, the efficacy is relatively limited and potential neurological deficits are easily overlooked.^7^ Butylphthalide targets vascular endothelium, reconstructs cerebral microcirculation, and promotes CVR injury recovery.^18^,^22^

It is reported that insufficient cerebral perfusion may be an important cause of cognitive impairment in patients with cerebral artery stenosis.^23^ When intracranial vascular stenosis reaches a certain degree, it can cause chronic ischemia and hypoxia of the brain tissue in the corresponding vascular innervation area, resulting in damage to cognitive function-related brain areas and leading to cognitive impairment.^24^ The MoCA scale is mainly used for rapid screening of cognitive dysfunction, with high sensitivity and a wide range of evaluation fields.^18^ The results of this study showed that the MoCA scores of the observation group were significantly higher than those of the control group.

Butylphthalide treatment of patients can, therefore, exert a strong anti-ischemic effect, significantly improve ischemic and hypoxic state of brain tissue, and thereby improve cognitive dysfunction, with a high neuroprotective effect.^25^,^26^ This study also found that butylphthalide effectively improves cerebral oxygen supply in patients and protects their neurological function. Butylphthalein inhibits the release of arachidonic acid, enhances nitric oxide expression and antioxidant enzyme activity, promotes free radical clearance, reduces inflammation and brain tissue damage, and effectively inhibits neuronal apoptosis.^27^ It has a good regulatory effect on brain energy metabolism, increasing blood flow in ischemic areas, inhibiting thrombosis, reducing infarct size, and promoting nerve defect recovery.^28^ Our results suggest that in stroke patients, treatment with butylphthalide restores microcirculation of ischemic brain tissue, and cerebrovascular reserve function. That leads to observed improvement in the cognitive impairment and neurological function, promoting the recovery of bodily functions and improving daily living abilities.^27^,^28^ Administering butylphthalide by injection can effectively avoid changes in transaminases, liver and kidney function, as well as prevent gastrointestinal reactions such as nausea and vomiting caused by oral preparations.^29^

### Limitations:

It is a single center retrospective study with the small sample size, and is, therefore, prone to selection bias. Additionally, neither group was randomly assigned, and baseline information may have been imbalanced and biased. The indicators monitored by transcranial Doppler ultrasonography may be influenced by human or technical factors. A longer follow-up period is needed to validate our results, as the impact of the two treatment methods on the long-term functional recovery of patients was not analyzed. Further high-quality research is needed to validate our conclusions.

## CONCLUSION

Butylphthalide in combination with routine treatment effectively improves cerebrovascular reserve function of patients with stroke caused by ICAS, promotes their neurological and cognitive dysfunction recovery, enhances daily living ability, has higher safety and significant therapeutic effect.

### Authors’ contributions:

**XL:** Conceived and designed the study.

**SQ**, **YW**, **XZ**, **YJ** and **BY:** Collected the data and performed the analysis.

**XL:** Was involved in the writing of the manuscript and is responsible for the integrity of the study.

All authors have read and approved the final manuscript.
